# Emollient bath additives for the treatment of childhood eczema (BATHE): multicentre pragmatic parallel group randomised controlled trial of clinical and cost effectiveness

**DOI:** 10.1136/bmj.k1332

**Published:** 2018-05-03

**Authors:** Miriam Santer, Matthew J Ridd, Nick A Francis, Beth Stuart, Kate Rumsby, Maria Chorozoglou, Taeko Becque, Amanda Roberts, Lyn Liddiard, Claire Nollett, Julie Hooper, Martina Prude, Wendy Wood, Kim S Thomas, Emma Thomas-Jones, Hywel C Williams, Paul Little

**Affiliations:** 1 Primary Care & Population Sciences, Faculty of Medicine, University of Southampton, Southampton SO16 5ST, UK; 2 Population Health Sciences, Bristol Medical School, University of Bristol, Bristol, UK; 3 Division of Population Medicine, School of Medicine, Cardiff University, Cardiff, UK; 4 Southampton Health Technology Assessments Centre, Wessex Institute, University of Southampton, Southampton, UK; 5 Centre of Evidence Based Dermatology, School of Medicine, University of Nottingham, Nottingham, UK; 6 Centre for Trials Research, College of Biomedical and Life Sciences, Cardiff, UK; 7 NIHR Research Design Service South Central, Primary Care and Population Sciences, University of Southampton, Southampton General Hospital, Southampton, UK

## Abstract

**Objectives:**

To determine the clinical effectiveness and cost effectiveness of including emollient bath additives in the management of eczema in children.

**Design:**

Pragmatic randomised open label superiority trial with two parallel groups.

**Setting:**

96 general practices in Wales and western and southern England.

**Participants:**

483 children aged 1 to 11 years, fulfilling UK diagnostic criteria for atopic dermatitis. Children with very mild eczema and children who bathed less than once weekly were excluded.

**Interventions:**

Participants in the intervention group were prescribed emollient bath additives by their usual clinical team to be used regularly for 12 months. The control group were asked to use no bath additives for 12 months. Both groups continued with standard eczema management, including leave-on emollients, and caregivers were given standardised advice on how to wash participants.

**Main outcome measures:**

The primary outcome was eczema control measured by the patient oriented eczema measure (POEM, scores 0-7 mild, 8-16 moderate, 17-28 severe) weekly for 16 weeks. Secondary outcomes were eczema severity over one year (monthly POEM score from baseline to 52 weeks), number of eczema exacerbations resulting in primary healthcare consultation, disease specific quality of life (dermatitis family impact), generic quality of life (child health utility-9D), utilisation of resources, and type and quantity of topical corticosteroid or topical calcineurin inhibitors prescribed.

**Results:**

483 children were randomised and one child was withdrawn, leaving 482 children in the trial: 51% were girls (244/482), 84% were of white ethnicity (447/470), and the mean age was 5 years. 96% (461/482) of participants completed at least one post-baseline POEM, so were included in the analysis, and 77% (370/482) completed questionnaires for more than 80% of the time points for the primary outcome (12/16 weekly questionnaires to 16 weeks). The mean baseline POEM score was 9.5 (SD 5.7) in the bath additives group and 10.1 (SD 5.8) in the no bath additives group. The mean POEM score over the 16 week period was 7.5 (SD. 6.0) in the bath additives group and 8.4 (SD 6.0) in the no bath additives group. No statistically significant difference was found in weekly POEM scores between groups over 16 weeks. After controlling for baseline severity and confounders (ethnicity, topical corticosteroid use, soap substitute use) and allowing for clustering of participants within centres and responses within participants over time, POEM scores in the no bath additives group were 0.41 points higher than in the bath additives group (95% confidence interval −0.27 to 1.10), below the published minimal clinically important difference for POEM of 3 points. The groups did not differ in secondary outcomes, economic outcomes, or adverse effects.

**Conclusions:**

This trial found no evidence of clinical benefit from including emollient bath additives in the standard management of eczema in children. Further research is needed into optimal regimens for leave-on emollient and soap substitutes.

**Trial registration:**

Current Controlled Trials ISRCTN84102309.

## Introduction

Childhood eczema (also known as atopic eczema or atopic dermatitis) is a common condition that can have a substantial impact on quality of life for children and their families.^1^ Guidelines suggest that complete emollient therapy forms the mainstay of treatment for eczema and should be used regularly with topical corticosteroids or topical calcineurin inhibitors, used in addition for flare-ups.^2^

Emollients are thought to act by providing a barrier over the skin, decreasing moisture loss, and protecting against skin irritants. Emollients are applied in one of three ways: leave-on, where emollients are directly applied to the skin; soap substitutes, where emollients are used instead of soap or other wash products; and bath additives, comprising oil or emulsifiers, or both designed to be added to bath water and thought to leave a film of oil over the skin. Some emollients can be used in more than one way. We therefore use the term “emollient bath additives” or “bath additives” rather than bath emollients to emphasise the differences between the three methods of application in recognition that products may have more than one method of application.

Although there is evidence for the need for leave-on emollients^3^ and widespread clinical consensus around soap substitutes, there is less agreement about the potential additional benefits of bath additives^4^ and a dearth of evidence on their effectiveness. Systematic reviews have found no evidence of effectiveness, and one small study suggested bath additives might actually worsen eczema outcomes.^5^ Bath additives are, however, widely prescribed at a cost of more than £23m ($33m; €26m) annually to the National Health Service in England.^6^

We determined both the clinical effectiveness and the cost effectiveness of including emollient bath additives in the standard management of eczema in children.

## Methods

This was a pragmatic, multicentre, randomised open label superiority trial with two parallel groups allocated in a 1:1 ratio comparing emollient bath additives in addition to standard eczema care compared with standard care alone for childhood eczema. We chose a pragmatic design^7^ that aimed to test whether bath additives offer additional benefit in real life eczema care rather than in ideal experimental conditions. The study was registered before recruitment of the first participant, and the study protocol has been published.^8^

Children eligible for the trial were aged 1 to 11 years and fulfilled UK diagnostic criteria for atopic dermatitis.^9^ We excluded children with inactive or very mild eczema over the past 12 months, defined as a score of 5 or less on the Nottingham eczema severity scale (scale from 3 to 15, where 3 to 8 is mild, 9 to 11 is moderate, and 12 to 15 is severe).^10^ We also excluded children who usually bathed less than once a week or whose carers were not willing to accept randomisation. Only one child was enrolled in each family.

Participants were recruited from 96 general practices in Wales and the west and south of England. We used the practices’ medical records to identify children with a recorded diagnosis of eczema and who had received one or more prescriptions for eczema in the past 12 months. General practice staff also recruited potential participants opportunistically. Parents or carers were asked to return a reply slip to the study team. A brief screening questionnaire included the UK diagnostic criteria for atopic dermatitis and the Nottingham eczema severity scale. A researcher telephoned parents or carers who expressed an interest in the study to confirm likely eligibility of children and to arrange a baseline appointment. Informed consent was sought at this time and baseline questionnaires completed. Subsequent questionnaires were completed online or by post with no further face-to-face contact between the participants and the trial team. Informed consent was received for trial participants before enrolment.

### Interventions

Participants in the intervention group were prescribed bath additives by their general practice and were asked to use them regularly for 12 months. We encouraged practices to issue the three bath additives most commonly prescribed in the UK: Oilatum (Glaxo SmithKline; 63% light liquid paraffin), Balneum (Allmarall; 85% soya oil), or Aveeno (Johnson & Johnson; no summary of product characteristics available). Other bath additives could be issued, with the exception of products containing antimicrobials, which we excluded as they have been shown to have a greater irritant effect than other bath additives.^11^ The control group were not prescribed bath additives and were asked not to use any bath additives for 12 months. Both groups were given standardised written advice on how to wash, including the use of leave-on emollient as a soap substitute. Both groups were advised to continue with standard eczema management, including regular leave-on emollients and topical corticosteroids when required. Ongoing clinical care was otherwise unchanged.

### Outcomes

The primary outcome was eczema severity measured by the patient oriented eczema measure (POEM) reported by parents or carers weekly over 16 weeks.^12^ ^13^ The POEM is a patient reported outcome, which scores symptoms over the previous week. It consists of seven questions that can be completed by the child’s parent or carer and provides a severity score on a scale from 0 to 28, where 0 to 2 is clear or almost clear, 3 to 7 is mild, 8 to 16 is moderate, and 17 to 28 is severe.^11^ The published minimal clinically important difference of the POEM is 3 points.^14^ ^15^ POEM was the only patient reported outcome measure for eczema to show validity and repeatability in a systematic review and has been adopted as the preferred patient reported outcome measure for eczema symptoms internationally.^16^ ^17^

The relapsing and remitting course of eczema means that repeated measures are a better reflection of effect than follow-up assessment at a single time point.

Secondary outcomes included eczema severity measured by POEM every four weeks from baseline to 52 weeks; disease specific quality of life at 16 weeks and one year, measured by dermatitis family impact^18^; generic quality of life at 16 weeks and one year, measured by child health utility-9D^19^; number of eczema exacerbations resulting in a primary healthcare consultation over one year (review of general practitioners’ (GPs’) notes); type (strength) and quantity of topical corticosteroid or topical calcineurin inhibitors prescribed over one year (review of GPs’ notes); resource use from parent or carer questionnaires and review of GPs’ notes; and other outcomes: adherence to treatment allocation (parent or carer report), and adverse effects, such as stinging, redness, slipping in the bath, or refusal to bathe (parent or carer report).

### Sample size

The sample size was calculated for repeated measures analysis of variance in weekly POEM scores over 16 weeks. Using weekly data from a similar population in the Softened Water Eczema Trial (SWET),^20^ we aimed to detect a mean difference of 2.0 (SD 7.0) points between intervention and control groups. Although the published minimal clinically important difference for POEM is 3 points,^14^ ^15^ we sought to detect a difference of 2 owing to the expectation of low POEM scores at baseline in a population recruited entirely through primary care. An α of 0.05 and power of 0.9, with a correlation between repeated measures of 0.70, gave a sample size of 338. Allowing for 20% loss to follow-up gave a total sample size of 423 participants.

Early data showed adherence to treatment allocation was achieved by approximately 80% of participants in both groups. Therefore, to allow a per protocol analysis with 90% power, in addition to the primary intention to treat analysis, we sought and obtained approval for an ethics amendment requesting permission to recruit an additional 68 participants, giving a revised target of 491 participants. No other changes to protocol occurred.

### Randomisation

Participants who provided consent were randomly allocated in 1:1 ratio to the intervention or control groups, stratified by coordinating centre (Southampton, Bristol, Cardiff). Randomisations were conducted at the end of the recruitment appointment, following completion of consent and the baseline questionnaire so that treatment allocation could not be known before study entry.

Randomisation was carried out using LifeGuide software hosted at the University of Southampton and automated to ensure concealment. As baseline appointments were sometimes remote from internet access, a back-up randomisation system involved phoning the trial manager. The unique participant identifier was then entered into a spreadsheet that allocated treatment on a 1:1 ratio, stratified by coordinating centre, from an MS Excel spreadsheet preprogrammed by the trial statistician. Thirty randomisations were conducted using this offline method.

It was not possible to make a convincing placebo for emollient bath additives, which add a greasy film to water, and participating families were therefore not blind to treatment allocation. As all outcomes were either reported by participants or collected on a clinical record review template, we did not mask clinical study officers or research nurses to allocation. Statisticians carrying out the analyses were blind to treatment allocation.

### Statistical analysis

Analysis was conducted according to CONSORT guidelines, following an analysis plan agreed in advance with the independent trial steering committee. We used descriptive statistics to compare baseline characteristics of trial participants by allocated group. The primary analysis for the total POEM score was performed using a multilevel mixed model framework with observations over time from weeks 1 to 16 (level 1) nested within participants (level 2). Our primary outcome is based on adjusted results, controlling for baseline POEM score, recruiting centre, and any significant confounders. We also report unadjusted results.

For all models, we analysed participants in the group to which they were randomised, regardless of their adherence to that allocation (intention to treat analysis). The only exception to this was the per protocol analysis, where analyses were carried out on the basis of bath additive use as reported by parents or carers.

The model used all the observed data and we made the assumption that missing POEM scores are missing at random given the observed data. The model included a random effect for centre (random intercept) and patient (random intercept and slope on time) to allow for differences between patients and between centres at baseline and between patients in the rate of change over time (if a treatment or time interaction was significant), and fixed effects for baseline covariates. We used an unstructured covariance matrix.

In the analysis of secondary outcomes, for the monthly POEM measure up to one year we used repeated measures analysis in line with that used for the primary outcome. For other secondary outcomes, we used linear regression for continuous outcomes if the assumptions were met. Otherwise we used non-parametric analyses. We used logistic regression for dichotomous outcomes and a suitable count model, as determined by goodness of fit measures, for count data. In all analyses we controlled for stratification variables and potential confounders. As set out in the statistical analysis plan, we carried out preplanned sensitivity analysis and exploratory subgroup analyses. For the economic evaluation we used resource use, cost and effectiveness data collected from participants, and reviews of GPs’ notes.

### Patient and public involvement

The James Lind Alliance Priority Setting Partnership for Eczema top 10 included priorities around bathing and washing and around the best ways to use emollients.^21^ This trial was funded following a topic suggestion form submitted through the National Institute for Health Research website, leading to a commissioned call.

The trial management group included an experienced patient and public involvement (PPI) co-applicant (AR) who participated in all phases of the trial design, including planning recruitment and recruitment materials. We also consulted members of the Centre of Evidence Based Dermatology patient panel at the trial design stage, and we sought additional PPI representation when planning how to disseminate findings. The independent trial steering committee included a PPI member. The results will be emailed to all trial participants and published on the trial website. The burden of the intervention was minimal, with many families already familiar with using bath additives with no difficulty.

## Results

**Fig 1:**
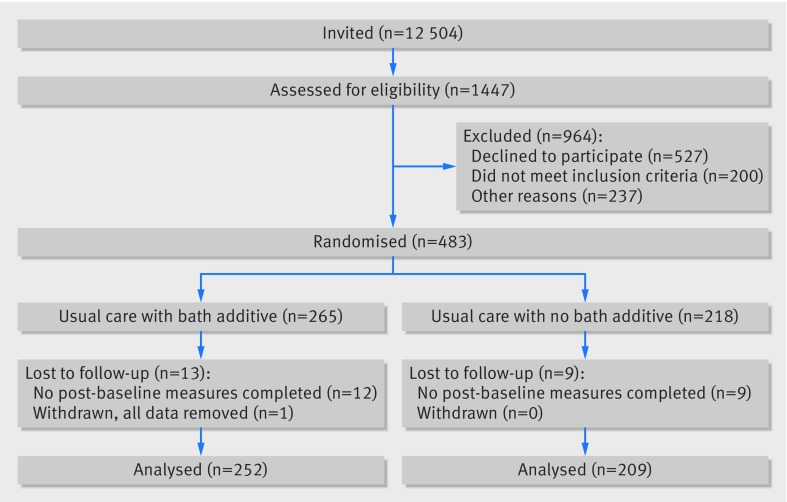
Flow of participants through trial

Participants were recruited between December 2014 and May 2016. Invitations were sent to the parents or carers of 12 504 children and 1451 responses were received. Of these, 920 expressed a willingness to be contacted and included a completed screening questionnaire. Overall, 662 met the eligibility criteria and were approached regarding participation, and 483 entered the trial. One carer subsequently withdrew permission, therefore analysis was carried out on data from 482 participants (n=264 intervention group, n=218 control group, fig 1).

Table 1 shows the characteristics of trial participants These were well balanced at baseline, although more participants were allocated to the bath additives group than to the no bath additives group.

**Table 1:** Baseline characteristics of trial participants. Values are numbers (percentages) unless stated otherwise

Characteristics	Bath additives (n=264)	No bath additives (n=218)	Total (n=482)
Mean (SD) age (years)	5.4 (2.9)	5.2 (2.9)	5.3 (2.9)
Sex:	n=264	n=218	n=482
Boys	138 (52)	100 (46)	238 (49)
Girls	126 (48)	118 (54)	244 (51)
Mean (SD) age of carers (years)	36.5 (6.5)	35.9 (6.7)	36.2 (6.5)
Sex of carers:	n=258	n=212	n-470
Male	11 (4)	12 (6)	23 (5)
Female	247 (96)	200 (94)	447 (95)
Ethnicity:	n=257	n=215	n=472
White	228 (86)	176 (82)	397 (84)
Black	6 (2)	9 (4)	15 (3)
Asian	15 (6)	16 (7)	31 (7)
Mixed race	10 (4)	9 (4)	19 (4)
Chinese	2 (1)	3 (1)	5 (1)
Other	3 (1)	2 (1)	5 (1)
Highest qualification:	n=257	n=213	n=470
Not answered	6 (2)	3 (1)	9 (2)
Degree or equivalent	106 (41)	90 (42)	197 (42)
Diploma or equivalent	56 (22)	37 (17)	95 (20)
A level	25 (10)	24 (11)	49 (10)
GSCE or O level	50 (19)	38 (18)	88 (19)
Other	12 (5)	16 (8)	29 (6)
None	2 (1)	5 (2)	7 (1)
Cost of living:	n=257	n=213	n=470
Not answered	6 (2)	3 (1)	9 (2)
Finding it a strain	11 (4)	3 (1)	14 (3)
Have to be careful	105 (41)	82 (38)	187 (40)
Able to manage	99 (39)	90 (42)	189 (40)
Quite comfortable	35 (14)	35 (16)	70 (15)
Prior belief in bath additives (1-9)*	5.1 (2)	4.8 (2)	5.0 (2)
Mean (SD) POEM score (0-28)	9.5 (5.7)	10.1 (5.8)	9.8 (5.8)
POEM scores†:	n=264	n=218	n=482
Mild (0-7)	114 (43)	73 (33)	187 (39)
Moderate (8-16)	119 (45)	114 (52)	233 (48)
Severe (17-28)	31 (12)	31 (14)	62 (13)
Median (IQR) DFIQ score (0-30)	2 (1-6)	3 (1-7)	3 (1-7)
Mean (SD) NESS score (3-15)	9.5 (2.3)	9.5 (2.3)	9.5 (2.3)
Mean (SD) CHU-9D score (utility values)	0.90 (0.1)	0.90 (0.1)	0.90 (0.1)

* Where 1 is not at all effective and 9 is very effective.

† 461/482 (96%) participants had completed at least one POEM after baseline and were included in the primary analysis, and 77% (370/482) completed more than 80% of time points for the primary outcome (12/16 weekly questionnaires to 16 weeks).

Parent or carer report of adherence to treatment allocation group at 16 weeks showed that 93% (216/233) of participants in the bath additives group used bath additives every time or more than half the time. Of those in the no bath additives group, 92% (176/191) said they used bath additives either never or less than half the time (table 2).

**Table 2:** Adherence to allocated treatment and frequency of bathing during 16 week primary outcome period. Values are numbers (percentages)

Treatment allocation	Bath additives	No bath additives
Use of bath additives:	(n=233)	(n=191)
Every time	172 (74)	14 (7)
>50% of the time	44 (19)	1 (0.5)
<50% of the time	15 (6)	9 (5)
Never	2 (1)	167 (87)
No of baths per week:	(n=221)	(n=176)
1-2	70 (32)	54 (31)
3-4	74 (33)	56 (32)
5-6	45 (20)	39 (22)
≥7	32 (14)	27 (15)

For frequency of bathing, parent or carer reports showed that 31% (124/397) of participants had fewer than three baths a week, 33% (130/397) had three or four baths a week, and 36% (143/397) had five or more baths a week.

Of participants allocated to receive bath additives, 45% (120/265) received Oilatum bath additive, 26% (68/265) Aveeno bath oil, 4.5% (12/265) Balneum bath oil, and 30% (79/265) another bath additive (14 received more than one different bath additive during the study).

**Fig 2:**
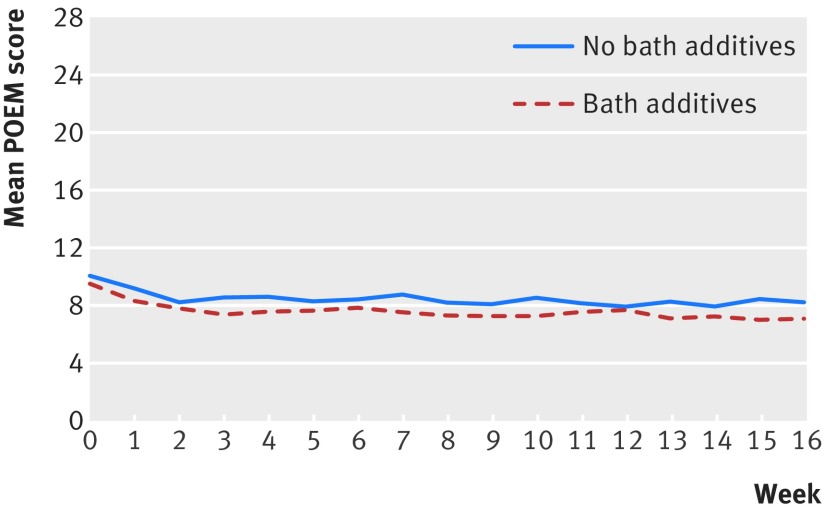
Patient oriented eczema measure (POEM) scores during 16 week primary outcome period

The baseline POEM score was 9.5 (SD 5.7) in the bath additives group and 10.1 (SD 5.8) in the no bath additives group. The mean POEM score over the 16 week period was 7.5 (SD 6.0) in the bath additives group and 8.4 (SD 6.0) in the no bath additives group (fig 2). No statistically significant difference was found in weekly POEM scores between the two groups over the 16 week period. After controlling for baseline severity and confounders (ethnic group, topical corticosteroid use, and soap substitute use) and allowing for the clustering of participants within centres and responses within participants over time, the POEM score in the no bath additives group was 0.41 points higher than in the bath additives group (95% confidence interval −0.27 to 1.10), which is substantially lower than the published minimal clinically important difference of 3 points.^14^ ^15^

Secondary analyses for differences between groups based on adherence to treatment allocation (per protocol analysis) similarly showed no statistically significant difference between the groups on POEM repeated measures over 16 weeks: “More than half” or “every time” versus “less than half the time” or “never” adjusted difference in mean POEM scores 0.32 (95% confidence interval −0.37 to 1.02).

No significant differences were found between groups in any of the secondary outcomes, such as POEM score over 52 weeks (adjusted difference in mean POEM score 0.75, 95% confidence interval −0.05 to 1.55), dermatitis family impact, generic quality of life (child health utility-9D), number of eczema exacerbations, and type or quantity of topical corticosteroid or topical calcineurin inhibitors prescribed over one year (table 3).

**Table 3:** Secondary outcomes by treatment allocation

Outcomes	Bath additives	No bath additives	Difference (95% CI)	
			Univariate	Adjusted
Mean (SD) repeated monthly measures over 52 weeks	7.3 (6.3)	8.4 (6.4)	0.99 (0.03 to 1.96)	0.75 (−0.05 to 1.55)
Median (interquartile range) disease specific quality of life:				
Baseline	2 (1-6)	3 (1-7)		
16 weeks	2 (0-5)	3 (1-7)	1.00 (0.09 to 1.91)	0.29 (−0.57 to 1.14)
52 weeks	2 (0-5)	2 (0-6)	0.00 (−0.93 to 0.93)	−0.29 (−1.36 to 0.79)
Mean (SD) generic quality of life*:				
Baseline	0.90 (0.1)	0.90 (0.1)	0.00 (−0.02 to 0.02)	
16 weeks	0.91 (0.1)	0.89 (0.1)	0.01 (−0.01 to 0.03)	
52 weeks	0.90 (0.1)	0.91 (0.1)	−0.01 (−0.03 to 0.01)	
Total No of TCS/TCI prescriptions	325	346		
Median (interquartile range) No of TCS/TCI prescriptions	0 (0-2)	1 (0-3)		
Median (interquartile range) No of exacerbations	1 (0-2)	1 (0-3)	1.33 (1.02 to 1.75)†	1.24 (0.96 to 1.60)†

TCS=topical corticosteroid; TCI=topical calcineurin inhibitor.

* Child health utility-9D.

† Relative risk (95% CI).

Prespecified exploratory subgroup analyses suggested the possibility of a small effect of bath additives among children aged less than 5 years, with the adjusted mean POEM score in the no bath additives group 1.29 (95% confidence interval 0.33 to 2.25) points higher than in the bath additives group. However, the upper limit of the confidence interval is below the now widely accepted POEM minimal clinically important difference of 3 points.

A statistically significant interaction effect was also seen by frequency of bathing as reported at 16 weeks. No statistically significant difference was found between children who bathed one to four times a week. In those who bathed five or more times a week, however, the POEM score was 2.27 points higher (0.63 to 3.91) in the no bath additives group. The upper confidence interval suggests there might be a small clinically meaningful benefit to bath additives in this group. But this group has only 77 in the bath additives group and 66 in the no bath additives group (table 4). No apparent difference was found in outcomes for those with moderate or severe eczema.

**Table 4:** Patient oriented eczema measure (POEM) scores during 16 week primary outcome period, by group and subgroup

Repeated measures	No (%)	Mean (SD)		Interaction term (95% CI)	Adjusted difference in mean POEM score* (95% CI)
		Bath additives	No bath additives		
Age (years):					
<5	256 (53)	6.99 (5.67)	9.09 (6.01)	−1.43 (−2.02 to −0.15)	1.29 (0.33 to 2.25)
≥5	226 (47)	7.97 (6.24)	7.52 (5.92)		−0.29 (−1.21 to 0.63)
Baseline eczema severity:					
Mild (0-7)	187 (39)	4.78 (4.26)	5.22 (4.58)	−0.05 (−1.14 to 1.05)	−0.07 (−1.08 to 0.95)
Moderate (8-16)	233 (48)	8.14 (5.54)	9.18 (5.46)		0.65 (−0.45 to 1.74)
Severe (17-28)	62 (13)	14.63 (6.16)	13.03 (6.92)		−1.16 (−3.62 to 1.32)
Use of leave-on emollient weekly:					
0-4 days	138 (29)	7.64 (6.68)	6.43 (5.42)	−0.02 (−2.05 to 2.01)	0.26 (−1.34 to 1.86)
5-7 days	344 (71)	8.61 (5.74)	7.93 (6.14)		0.69 (−0.39 to 1.76)
Topical corticosteroid use:					
Any	241 (51)	8.40 (6.19)	9.35 (6.21)	0.52 (−1.35 to 2.40)	1.22 (−0.18 to 2.63)
None	234 (49)	6.63 (5.64)	7.39 (5.66)		0.58 (−0.64 to 1.81)
Frequency of bathing at 16 weeks:					
1-4 times/week	255 (64)	7.93 (5.94)	8.00 (5.82)	2.14 (0.21 to 4.07)	−0.26 (−1.38 to 0.87)
≥5 times/week	143 (36)	6.30 (5.70)	8.75 (6.12)		2.27 (0.63 to 3.91)
Prior belief in bath additives:					
Low (1-3)	106 (29)	7.93 (6.10)	9.27 (6.25)	0.85 (−0.52 to 2.21)	1.17 (−0.78 to 3.13)
Moderate (4-6)	158 (44)	8.37 (6.06)	8.68 (6.02)		−0.16 (−1.77 to 1.45)
High (7-9)	97 (27)	5.70 (5.06)	7.09 (6.05)		1.80 (0.04 to 3.56)
Use of soap substitute at 16 weeks:					
Any	89 (21)	8.09 (6.10)	9.31 (5.88)	1.30 (−0.97 to 3.57)	1.72 (−0.44 to 3.88)
None	340 (79)	7.17 (5.82)	7.99 (5.87)		0.36 (−0.63 to 1.35)

* Adjusted for baseline severity, ethnic group, steroid use, and soap substitute use and allowing for clustering of patients within centres and responses within patients over time.

Adverse effects were noticeably similar in both groups, despite slips in the bath, stinging, or redness being common side effects reported in the summary of product characteristics for emollient bath additives. Over the first 16 weeks, 35% in the bath additives group and 35% in the no bath additives group reported at least one adverse event on weekly questionnaires (table 5), with no statistically significant difference between the groups (odds ratio 1.40, 95% confidence interval 0.79 to 2.47).

**Table 5:** Adverse events by treatment allocation. Values are numbers (percentages)

Adverse events by follow-up	Bath additives (n=252)	No bath additives (n=209)
16 weeks:		
Slipping in bath	44 (17)	52 (25)
Stinging	4 (2)	4 (2)
Redness	35 (14)	48 (23)
Refusal to bathe	21 (8)	25 (12)
52 weeks:		
Slipping in bath	56 (22)	63 (30)
Stinging	7 (3)	4 (2)
Redness	44 (17)	61 (29)
Refusal to bathe	30 (12)	31 (15)

For the economic evaluation we followed a prespecified analysis plan and explored resource utilisation from the perspective of the national health service and family. The mean annual costs to the NHS were estimated at £180.50 (SD £237.0) for the bath additives group and £166.12 (SD £293.0) for the no bath additives group. The annual results for quality of life years were 0.91 (SD 0.1) for the bath additives group and 0.90 (SD 0.1) for the no bath additives group. The difference in cost means was £14.38 (95% confidence interval −£33.45 to £62.21) and in quality adjusted life years means was 0.00 (−0.01 to 0.02). The costs borne by families showed an annual higher spend within the no bath additives group of £51.37 (95% confidence interval −£15.74 to £118.49) and the adjusted difference was £47.56 (−£18.07 to £113.19), none of which were statistically significant. For the economic analysis we found no benefits that could be used to consider the intervention cost effective. Full data on resource use (review of GPs’ notes and parent or carer report) and cost effectiveness analysis will be published in the National Institute for Health Research health and technology assessment journals library.

## Discussion

This trial provides strong evidence that emollient bath additives provide minimal or no additional benefit beyond standard eczema care in the management of eczema in children.

The BATHE trial was an adequately powered trial, with high follow-up rates and good adherence to trial intervention allocations. The study has strong external validity as it was pragmatic in design to reflect normal practice, and, despite the relatively low response rate, participants were broadly reflective of children with eczema seen in primary care. We used a participant reported outcome measure with good validity that has been accepted by international consensus.^15^ A participant reported outcome could be biased in favour of finding a positive effect of trial intervention owing to a perception of benefits of treatment. However, the negative result of the trial suggests that this was not the case.

This trial was large; previous reviews of the literature have not been able to draw conclusions owing to the small size of existing trials.^5^

We cannot exclude the possibility of a small benefit among children bathing more than five times a week or among children aged less than 5 years, but differences are sufficiently small to be unlikely to be clinically useful. Furthermore, caution is needed in interpreting these underpowered subgroup analyses as statistically significant results might arise because the data have been tested multiple times rather than because a genuine difference exists between the groups.

These findings are timely for clinicians and prescribing advisers, as prescribing guidelines vary widely in their advice on the use of bath additives,^22^ and pressure on budgets has led to formularies becoming increasingly restrictive. Reviews have estimated that bath additives might contribute to up to one third of the costs of eczema in the United Kingdom.^4^ Our findings provide evidence that can contribute to effective prescribing in this area, where there is currently little research evidence to guide decision making. These findings are also useful for families with members who have eczema as they have more certainty about directing their efforts towards more effective treatments.

Our findings are only relevant to the use of emollient bath additives. More research is needed into optimal regimens for other emollients, although there is strong evidence that regular use of leave-on emollients prevents flare-ups in eczema,^3^ and there is widespread clinical consensus around the role of emollients as soap substitutes.

**What is already known on this topic**

There are three methods of application of emollients; leave-on emollients, soap substitutes and emollient bath additives
Although evidence supports the use of leave-on emollients and there is clinical consensus around soap substitutes, less agreement exists about the benefits of emollient bath additives to treat eczema in children
The effectiveness of emollient bath additives to treat childhood eczema has not been assessed owing to a lack of adequately powered trials

**What this study adds**

This large, pragmatic randomised controlled trial of children with eczema (aged 1-11 years) found no evidence of a clinically meaningful benefit from emollient bath additives, when used in addition to standard eczema management
Questions remain about optimal regimens for leave-on treatments, soap substitutes, and frequency of bathing in eczema treatment

## Notes

Contributors: MS, MJR, NAF, PL, KST, HCW, AR, BS, MC, and WW obtained research funding. All authors contributed to the development of the protocol and management of the study. KR led the day-to-day management of the study, supported by MS, MJR, NAF, WW, and PL. MS, KR, and BS drafted the paper with contributions from all authors. MS is the guarantor. All authors read and approved the final manuscript. The views and opinions expressed therein are those of the authors and do not necessarily reflect those of the Health Technology Assessment Programme, National Institute for Health Research, National Health Service, or Department of Health.

Ethical approval: BATHE was approved by the Newcastle & North Tyneside research ethics committee 1 (14/NE/0098).

Data sharing: No additional data available.

Transparency: The guarantor (MS) affirms that the manuscript is a honest, accurate, and transparent account of the study bring reported; that no important aspects of the study have been omitted; and that any discrepancies from the study as planned (and, if relevant, registered) have been explained.
